# Investigation of TbfA in *Riemerella anatipestifer* using plasmid-based methods for gene over-expression and knockdown

**DOI:** 10.1038/srep37159

**Published:** 2016-11-15

**Authors:** MaFeng Liu, MengYi Wang, DeKang Zhu, MingShu Wang, RenYong Jia, Shun Chen, KunFeng Sun, Qiao Yang, Ying Wu, XiaoYue Chen, Francis Biville, AnChun Cheng

**Affiliations:** 1 Institute of Preventive Veterinary Medicine, Sichuan Agricultural University, Chengdu, Sichuan 611130, P.R. China; 2 Avian Disease Research Center, College of Veterinary Medicine of Sichuan Agricultural University, Chengdu, Sichuan 611130, P.R. China; 3 Key Laboratory of Animal Disease and Human Health of Sichuan Province, Sichuan Agricultural University, Chengdu, Sichuan 611130, P. R. China; 4 Unité des Infections Bactériennes Invasives, Département Infection et Epidémiologie, Institut Pasteur, Paris, France

## Abstract

*Riemerella anatipestifer* is a duck pathogen that has caused serious economic losses to the duck industry worldwide. Despite this, there are few reported studies of the physiological and pathogenic mechanisms of *Riemerella anatipestifer* infection. In previous study, we have shown that TonB1 and TonB2 were involved in hemin uptake. TonB family protein (TbfA) was not investigated, since knockout of this gene was not successful at that time. Here, we used a plasmid based gene over-expression and knockdown to investigate its function. First, we constructed three *Escherichia*-*Riemerella anatipestifer* shuttle vectors containing three different native *Riemerella anatipestifer* promoters. The shuttle plasmids were introduced into *Riemerella anatipestifer* ATCC11845 by conjugation at an efficiency of 5 × 10^−5^ antibiotic-resistant transconjugants per recipient cell. Based on the high-expression shuttle vector pLMF03, a method for gene knockdown was established. Knockdown of TbfA in *Riemerella anatipestifer* ATCC11845 decreased the organism’s growth ability in TSB medium but did not affect its hemin utilization. In contrast, over-expression of TbfA in *Riemerella anatipestifer* ATCC11845*ΔtonB1ΔtonB2*. Significantly promoted the organism’s growth in TSB medium but significantly inhibited its hemin utilization. Collectively, these findings suggest that TbfA is not involved in hemin utilization by *Riemerella anatipestifer*.

*Riemerella anatipestifer (R. anatipestifer*, RA) is a gram-negative bacterium belonging to the family *Flavobacteriaceae*^1^. RA does not encode the genes *hemF, hemY,* and *hemG,* which are essential for *de novo* synthesis of hemin^2^. Usually, RA was cultured on the medium containing hemin source, such as sheep blood plate. RA can infect most types of poultry, including ducks, chicken, geese and turkeys^3^. To date, at least 21 serotypes have been described^4^,^5^. Among them, serotypes 1, 2, 3, 5, and 15 are the major pathogens affecting the duck industry. No cross protection has been found among these serotypes^5^,^6^. RA infection produces mortality and morbidity rates ranging between 10 and 30%, but mortality rates as high as 75% have been reported in infected duck farms^7^. Presently, little is known about the molecular mechanism underlying RA pathogenesis.

Efforts have been made to understand the mechanisms of virulence employed by this organism. Several virulence-associated factors have been proposed, including CAMP^8^, OmpA^9^, TbdR1^10^, siderophore-interacting protein (Sip)^11^, M949-1556^12^, AS87_03730^13^ and the TonB system^14^. The bulk of the evidence that these factors play a role in virulence is suggestive, based on gene knockout studies, observed symptoms and median lethal dose (LD50) values. However, gene knockout can change the expression of downstream genes, which is known as the polar effect. When this occurs, shuttle vectors can be utilized to complement a knockout strain to better estimate the effect caused by the knockout.

In a previous study, we identified the functions held by ExbB-ExbD-TonB (TonB1 system) encoded by *RA0C_1191-RA0C_1192*-*RA0C_1193*, ExbB-ExbD-ExbD-TonB (TonB2 system) encoded by *RA0C_1212*-*RA0C_1211*-*RA0C_1210*-*RA0C_1209*, and a TonB family protein (TbfA) encoded by RA0C_0334, in *R. anatipestifer* ATCC11845^2^. Only *tbfA* is not linked with *exbB*-*exbD* genes (whereas *tonB1* and *tonB2* are), and hence it would unlikely be involved in iron/hemin transport.

There is only approximately 10.00% identity between TonB1 and TonB2, 10.65% identity between TonB1 and TbfA, and 34.71% identity between TonB2 and TbfA. Hemin transport is nearly abolished only when both *tonB1* and *tonB2* are deleted, suggesting that both TonB1 and TonB2 are involved in hemin transport in *R. anatipestifer* and that they are functionally redundant^2^. At that time, knockout of TbfA was not successful and it was hypothesized to be an essential gene^2^, and the technology required to do so was not available.

To investigate the function of TbfA, we first constructed a series of *E. coli*-*R. anatipestifer* shuttle vectors based on plasmid pMM47. A^15^ and the putative replication region of plasmid pRA0726 of *R. anatipestifer* 0726^16^. These vectors could be used for gene over-expression and gene knockdown in *R. anatipestifer* strains. Using these methods, we showed that the TbfA protein in *R. anatipestifer* ATCC11845 is not involved in hemin utilization but is required for optimal growth.

## Results

### Generation of stable replicating shuttle vector for *R. anatipestifer*

To construct the shuttle plasmid, we synthesized the replication region of plasmid pRA0726 from *R. anatipestifer* 0726 (GenBank accession no JF268688)^16^. We then generated shuttle vectors by amplifying this replication region, including 497 bp of its upstream region, and using it to replace the replication region of plasmid pMM47. A (as described in the *Materials and Methods* section). The resulting vector, pLMF01 (Fig. S1), could be mobilized from *E. coli* S17-1 to *R. anatipestifer* ATCC11845 with transfer frequencies of approximately 5 × 10^−5^ per recipient cell. This plasmid could also be transferred to RA-CH-1 and RA-CH-2 with transfer frequencies of approximately 10^−6^ per recipient cell.

The stability of the pLMF01 shuttle plasmid was evaluated in the *R. anatipestifer* ATCC11845 strain. To accomplish this, the *R. anatipestifer* ATCC11845 pLMF01 strain was grown through 10 generations in the absence or presence of cefoxitin. Ten single bacterial clones from the plate with or without antibiotic were picked to identify the cefoxitin resistance gene (*cfx*) by PCR. To excluded the plasmid was integrated into the chromosome, the plasmids were also extracted from *R. anatipestifer* ATCC11845 pLMF01 and submitted to PCR and restriction analysis. The results showed that the *cfx* gene could be amplified from all isolated clones and isolated plasmids pLMF01, demonstrating that this plasmid can stably replicate in *R. anatipestifer*(data not shown). The plasmid copy number of pLMF01 in *R. anatipestifer*ATCC11845 was evaluated by the method described by Lee *et al.*^17^. The result shown that the plasmid copy number was 20 to 30 per *R. anatipestifer*ATCC11845.

### Construction of plasmids for different expression in *R. anatipestifer*

Gene expression can vary to a great extent according to the strength of a promoter. Performing gene complementation assays that achieve different levels of gene expression thus requires the use of plasmids that enable low, medium or high expression of a cloned gene. To create such plasmids in the current study, three distinct promoter regions were predicted and selected based on RNA-seq data (unpublished results). We amplified the promoters of the genes *B739_0921* (rpkm:27503, high transcriptional activity), *B739_0973* (rpkm:2236, medium transcriptional activity) and *B739_0889* (rpkm:993, low transcriptional activity) from RA-CH-1 and used them to replace the *ermF* promoter in plasmid pLMF01, giving the plasmids pLMF03, pLMF04 and pLMF05, respectively (Fig. S1). To gauge the transcriptional activities of the resulting plasmids, the *R. anatipestifer tonB1* gene was first used to proof-of-principle. The *tonB1* gene was amplified and cloned into all three vectors, as described in the *Materials and Methods* section. Then, the three recombinant plasmids were introduced into the RA ATCC11845 *∆tonB1* strain. The transcription level of *tonB1* under the different promoters were evaluated by qRT-PCR and TonB1 protein expression levels under the different promoters were evaluated by western blotting using a specific antibody of TonB1^2^. qRT-PCR results showed that the transcription level of *tonB1* in RA ATCC11845 *∆tonB1* pLMF03*::tonB1* is more than 20-folds compare to that of RA ATCC11845 pLMF03, the transcription level of *tonB1* in RA ATCC11845 *∆tonB1* pLMF04*::tonB1* and RA ATCC11845 *∆tonB1* pLMF05*::tonB1* is about 0.8-fold and 0.4-fold, respectively, compare to that of RA ATCC11845pLMF03 (Fig. S2). Western-blot results indicated that all three plasmids described here were capable of expressing the gene in *R. anatipestifer*. For plasmid pLMF03, which contained the P_739_0921_ promoter region, there was greater TonB1 expression than for plasmid pLMF04, which contained the P_B739_0973_ promoter region. For plasmid pLMF05, which contained the P_B739_0889_ promoter region, there was lower expression than with plasmids pLMF04 and pLMF03 (Fig. S3).

**Figure 1:**
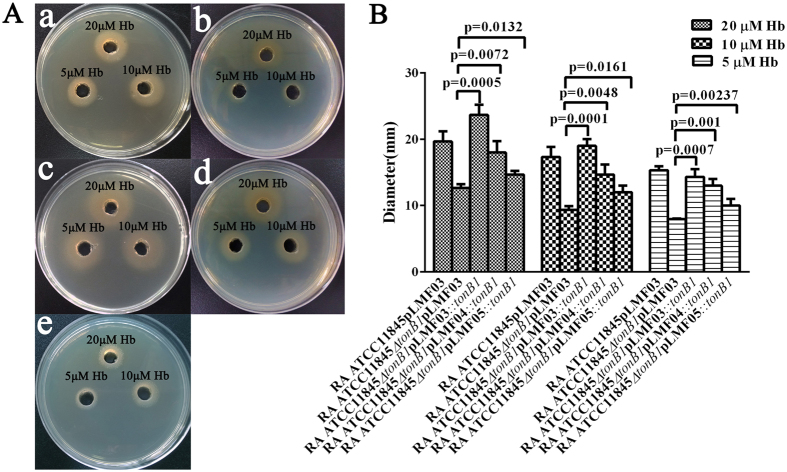
Complementation of RA ATCC11845 *∆tonB1* for hemin utilization. The strains RA ATCC11845pLMF03 (a), RA ATCC11845 Δ*tonB1*pLMF03 (b), RA ATCC11845Δ*tonB1*pLMF03::*tonB1* (c), RA ATCC11845 Δ*tonB1*pLMF04::*tonB1* (d), and RA ATCC11845 Δ*tonB1*pLMF05::*tonB1* (e) were tested for hemin utilization efficiency on LB plates as described in the *Materials and Methods* section (**A**). After 24 h of growth, the diameter of the zone of turbidity in each well was measured in quadruplicate for each plate, and the mean diameter was calculated (**B**). The results are expressed as the mean ± SD of the diameters (in mm) obtained from three independent experiments. The data were analyzed using Student’s t-test.

To further ensure the plasmids can be used for complementation, we also used *R. anatipestifer* ATCC11845 *∆tonB1* as proof-of-principle, since *R. anatipestifer* ATCC11845 TonB1 mutant was defective in hemin utilization from bovine hemoglobin. Previously, it was reported by our group that bovine hemoglobin were insufficient to support the growth of RA. This statement was incorrect, since we did not use high quality of bovine hemoglobin source to perform the experiment at that time. The evaluation was performed on the LB plate in the presence of different concentration of bovine hemoglobin. The results showed that plasmids pLMF03*::tonB1*, pLMF04*::tonB1*, and pLMF05*::tonB1* all augmented the growth of *R. anatipestifer* ATCC11845 *∆tonB1* in the presence of hemoglobin (Fig. 1A). After measuring the diameter of colony growth in each well used to culture *R. anatipestifer* ATCC11845 *∆tonB1*, we found that plasmid pLMF03*::tonB1* had significantly higher complementation ability than the others (Fig. 1B). This result was in accordance with the results related to the TonB1 expression levels measured in the *R. anatipestifer* ATCC11845 *∆tonB1* strains harboring plasmids pLMF03*::tonB1*, pLMF04*::tonB1*, or pLMF05*::tonB1*.

**Figure 2:**
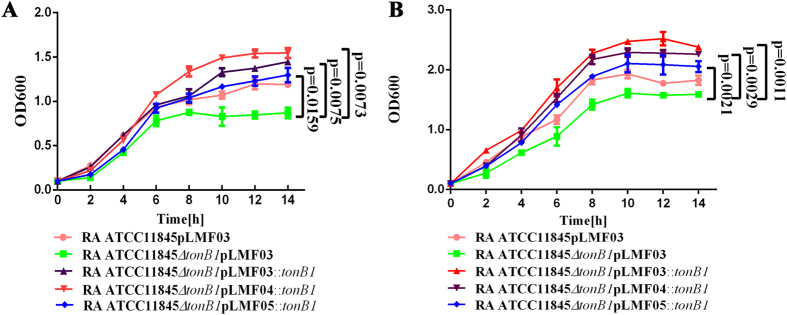
Growth curves for RA ATCC11845, RA ATCC11845Δ*tonB1* and the corresponding complementation strains in TSB medium (**A**) and TSB medium with 10 μM bovine hemoglobin (**B**). Cells were grown in 20 ml TSB medium or TSB with 10 μM bovine hemoglobin at 37 °C starting at OD600 = 0.1. OD600 values were then measured every 2 h for 14 h. The data were analyzed using two-way ANOVA. The error bars represent the standard deviation of three independent experiments.

We also performed complementation of *R. anatipestifer* ATCC11845 *∆tonB1* in TSB liquid medium with or without hemoglobin. Disruption of *tonB1* decreased the growth of *R. anatipestifer* ATCC11845 in TSB liquid medium with or without hemoglobin (Fig. 2). Additionally, complementation with any of the *tonB1* expressing plasmids was sufficient to restore *R. anatipestifer* ATCC11845 growth in TSB medium with or without bovine hemoglobin (Fig. 2).

### The shuttle plasmid based *tbfA* gene knockdown in *R. anatipestifer* ATCC11845

Gene knockout is useful for studying the functions of non-essential genes, whereas gene knockdown enables investigations into the functions of essential genes, such as *tbfA* gene of *R. anatipestifer* ATCC11845^2^. To produce high levels of antisense mRNA, the entire coding region of the *tbfA* gene was directionally cloned behind the promoter in the pLMF03 vector in the reverse orientation, such that the antisense strand was transcribed^18^. RT-PCR was used to demonstrate that the *tbfA* antisense strand was transcribed in RA ATCC11845 pLMF03::*tbfA*-antisense, but not in RA ATCC11845 pLMF03 (Fig. S4). Western blotting also was used to evaluated the expression of TbfA protein in *R. anatipestifer* ATCC11845 pLMF03::*tbfA*-antisense using the antibody specific to TbfA. Unfortunately, even in wild-type *R. anatipestifer* ATCC11845, TbfA protein could not be detected (data not shown). This result might be explained by the very low endogenous transcription levels of the *tbfA* structural gene (rpkm:17, unpublished data). Nevertheless, the decreased expressed of TonB1 in *R. anatipestifer* ATCC11845 pLMF03::*tonB1*-antisense was used to demonstrate this method working well in *R. anatipestifer* ATCC11845 (Fig. S5).

**Figure 3:**
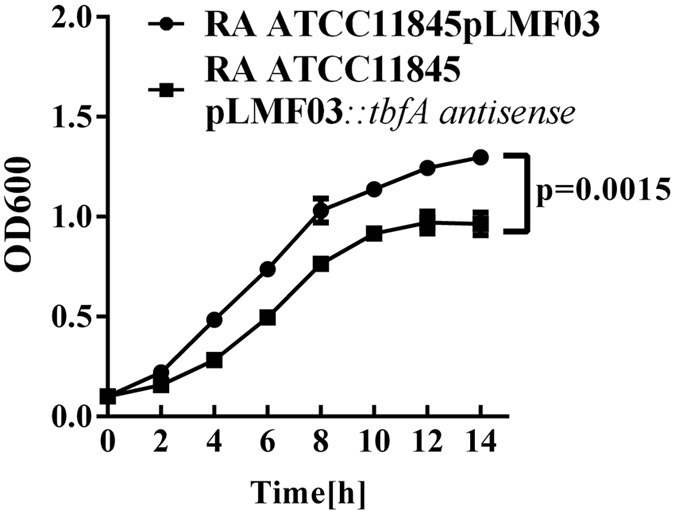
Growth curves for RA ATCC11845 pLMF03 and RA ATCC11845 pLMF03::*tbfA-*antisense in TSB medium. Cells were grown in 20 ml TSB medium at 37 °C starting at OD600 = 0.1. OD600 values were measured every 2 h for 14 h. The bacterial growth rate of RA ATCC11845 pLMF03::*tbfA*-antisense was significantly slower than that of RA ATCC11845 pLMF03 (p = 0.0015). The data were analyzed using two-way ANOVA. The error bars represent the standard deviation of three independent experiments.

To evaluate the physiological effects of decreased expression of TbfA protein, we compared the growth of *R. anatipestifer* ATCC11845 pLMF03 and *R. anatipestifer* ATCC11845 pLMF03::*tbfA*-antisense in TSB medium. As shown in Fig. 3, the introduction of the pLMF03::*tbfA*-antisense plasmid significantly decreased the growth of *R. anatipestifer* ATCC11845 in TSB medium.

### Knockdown of TbfA does not affect hemin utilization in *R. anatipestifer* ATCC11845 and *R. anatipestifer* ATCC11845*∆tonB1∆tonB2*

Knockout of *tonB1* and/or *tonB2* has been shown to significantly decrease the ability of *R. anatipestifer* ATCC11845 to transport hemin^2^. This led us to wonder whether *tbfA* knockdown would decrease the ability of *R. anatipestifer* ATCC11845 to transport hemin. To answer this question, we performed a hemin utilization assay using the strains *R. anatipestifer* ATCC11845 pLMF03 and *R. anatipestifer* ATCC11845 pLMF03*::tbfA-*antisense as described in the *Material and Methods* section. The results showed that there was no significant difference between the growth characteristics of *R. anatipestifer* ATCC11845 pLMF03 and *R. anatipestifer* ATCC11845 pLMF03*::tbfA*-antisense in the test wells (Fig. S6). Considering that TonB1 and TonB2 have redundant functions with regard to hemin uptake, we also performed hemin utilization assays using the *R. anatipestifer* ATCC11845 *∆tonB1∆tonB2* pLMF03 and *R. anatipestifer* ATCC11845 *∆tonB1∆tonB2* pLMF03*::tbfA*-antisense strains. There were no significant differences in the growth characteristics of the *R. anatipestifer* ATCC11845 *∆tonB1∆tonB2* pLMF03 and *R. anatipestifer* ATCC11845 *∆tonB1∆tonB2* pLMF03*::tbfA*-antisense strains (Fig. S6). Based on these results, we deduced that TbfA is not involved in hemin uptake in *R. anatipestifer* ATCC11845.

### Over-expression of TbfA in *R. anatipestifer* ATCC11845 and *R. anatipestifer* ATCC11845*∆tonB1∆tonB2* significantly inhibits hemin utilization

**Figure 4:**
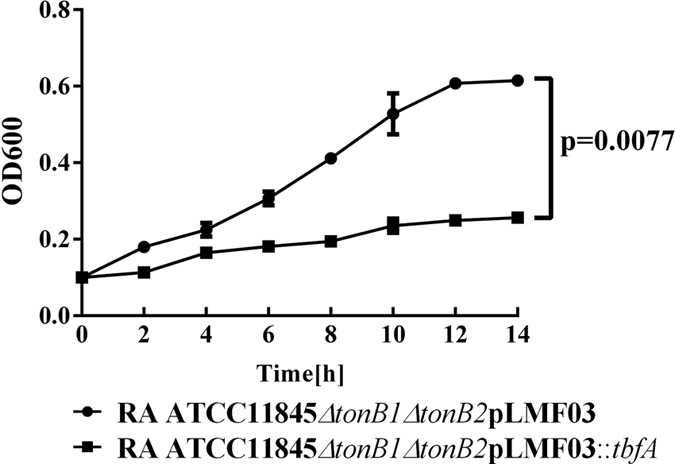
Growth curves for RA ATCC11845 *∆tonB1∆tonB2*pLMF03 and RA ATCC11845 *∆tonB1∆tonB2*pLMF03*::tbfA* in TSB medium. Cells were grown in 20 ml TSB medium at 37 °C starting at OD600 = 0.1. OD600 values were measured every 2 h for 16 h. The bacterial growth rate of RA ATCC11845 *∆tonB1∆tonB2*pLMF03 was significantly slower than that of RA ATCC11845 *∆tonB1∆tonB2*pLMF03*::tbfA* p = 0.0015). The data were analyzed using two-way ANOVA. The error bars represent the standard deviation of three independent experiments.

**Figure 5:**
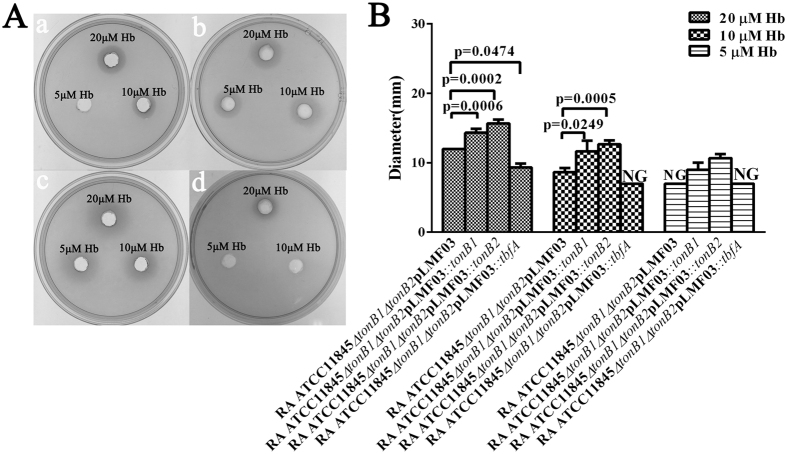
Hemin transport activity assays for RA ATCC11845 *∆tonB1∆tonB2*pLMF03, RA ATCC11845 *∆tonB1∆tonB2*pLMF03*::tonB1,* RA ATCC11845 *∆tonB1∆tonB2*pLMF03*::tonB2* and RA ATCC11845 *∆tonB1∆tonB2*pLMF03*::tbfA.* The above strains were tested for hemin utilization efficiency on LB plates as described in the *Materials and Methods* section. After 48 h of growth, the diameter of the zone of turbidity in each well was measured in quadruplicate for each plate, and the mean diameter was calculated. The results are expressed as the mean ± SD of the diameters (in mm) obtained for the three plates. NG: No growth in the well. The data were analyzed using Student’s t-test.

To further ensure that TbfA is not involved in hemin uptake, we over-expressed TbfA in *R. anatipestifer* ATCC11845*∆tonB1∆tonB2* and evaluated the strain’s hemin uptake activity. Over-expression of TbfA protein in *R. anatipestifer* ATCC11845*∆tonB1∆tonB2* promoted the growth of this strain in TSB medium (Fig. 4), whereas the hemin uptake activity of this strain was significantly inhibited under these conditions (Fig. 5). In contrast, the strain’s hemin uptake activity was significantly strengthened upon the expression of TonB1 or TonB2 (Fig. 5). Furthermore, hemin uptake activity was significantly inhibited by the over-expression of TbfA in wild-type RA ATCC11845 (Fig. S7).

## Discussion

In gram-negative bacteria, the TonB system harnesses the proton motive force to power outer membrane transporters. The TonB system modulates a number of outer membrane active transporters, each specific for one or more substrates^19^. TonB is the main iron-siderophore complex acquiring-machinery in Gram-negative bacteria^20^,^21^. The transportation of hemin, vitamin B12, nickel complexes and carbohydrates also require TonB activity^22^,^23^,^24^. We have previously shown that disruptions in the *tonB1* and/or *tonB2* genes seriously damages the hemin and iron uptake processes in *R. anatipestifer* ATCC11845^2^. However, the function of TbfA was not identified due to unability to knock it out.

To investigate the function of TbfA using gene over-expression and knockdown method, we first created shuttle plasmids that can replicate stable in *R. anatipestifer.* Subsequently, three different promoters were cloned in to the shuttle plasmid. The gene *tonB1* was inserted the shuttle plasmids and the promoter strength was evaluated by detecting the transcription of *tonB1* through qRT-PCR and by detecting the expression of TonB1 in *R. anatipestifer tonB1* mutant strain. Derivatives of pLMF03, pLMF04 and pLMF05 plasmids expressing TonB1 were shown to complement the *R. anatipestifer ∆tonB1* mutant strain in hemin uptake activity. The levels of complementation were in good accordance with the strengths of the promoters contained in these plasmids. These data indicated that all these established genetic tools were able to use to investigate the function of TbfA.

In the case of TbfA, the ability to interfere with gene expression using strategies such as the introduction of antisense RNA may provide a useful alternative to studying gene function, as this method does not completely abolish gene expression. Indeed, antisense RNA strategies have proven optimal for other bacteria^18^,^25^,^26^. TbfA knockdown seriously damaged the growth of *R. anatipestifer* in TSB medium. In contrast, TbfA knockdown did not affect hemin uptake in *R. anatipestifer* ATCC11845. Moreover, TbfA knockdown did not affect the residual hemin uptake activity of a *R. anatipestifer* ATCC11845 *∆tonB1∆tonB2* mutant. Collectively, these results strongly suggest that TbfA is not involved in hemin uptake in this species.

To strengthen this conclusion, we also over-expressed TbfA in *R. anatipestifer* ATCC11845 *∆tonB1∆tonB2* to determine whether this complementation would restore full hemin transport activity in this mutant. In contrast with TonB1 and TonB2, the over-expression of TbfA did not restore hemin transport activity in *R. anatipestifer* ATCC11845 *∆tonB1∆tonB2*. Surprisingly, over-expression of TbfA instead significantly inhibited hemin uptake activity in *R. anatipestifer* ATCC11845 *∆tonB1∆tonB2* and *R. anatipestifer* ATCC11845. One possible explanation for this result is that TbfA competes with TonB1 and TonB2 for the use of their cognate ExbB-ExbD systems, thus decreasing hemin uptake activity. This hypothesis is now under investigated in our lab. Additionally, the low identities between TonB2 and TonB2 (10%), TonB1 and TbfA (10.65%), and TonB2 and TbfA (34.71%) would partly explain the specificities of different TonB proteins in substrate transportation.

Although TbfA activity was not associated with the hemin transport system, it was still absolutely required for *R. anatipestifer* ATCC11845 viability. As a consequence, we concluded that TbfA activity is required for a transport process not related to hemin uptake. Identification of this transport process is still under investigation. It is also interesting to note that *R. anatipestifer* ATCC11845 *∆tonB1∆tonB2* retains hemin uptake activity, despite that *tbfA* does not function in hemin uptake. These results suggest that there are additional TonB-like proteins or an unknown hemin uptake system in *R. anatipestifer*.

In summary, in the current study, techniques to facilitate gene complementation and knockdown in *Riemerella* were developed, offering the possibility to genetically analyze bacterial species in the genus *Riemerella*. These tools will provide the basis for developing new approaches towards understanding the mechanisms underlying the pathogenesis of *Riemerella* infection.

## Materials and Methods

### Bacterial strains and plasmids

The bacterial strains and plasmids used in this study are shown in Table S1. The *Escherichia coli*–*Capnocytophaga canimorsus* shuttle plasmid pMM47. A was generously provided by Guy R. Cornelis, Biozentrum der Universität Basel, CH-4056 Basel, Switzerland.

### Media and growth conditions

Bovine hemoglobin was obtained from Sigma Chemical (Sigma, China). Hemoglobin concentration was calculated on the basis of the hemin monomer. Hemoglobin solutions were filter sterilized with 0.45-μm Millipore filters. *E. coli* strains were grown on LB medium (Sigma-Aldrich, Product Number: L3522) aerobically at 37 °C. The solid media contained 1.5% Difco agar. *R. anatipestifer* strains were cultured on LB agar supplemented with 5% sheep blood or in TSB liquid medium (Solarbio, China) at 37 °C. When necessary, the medium was supplemented with appropriate antibiotics at the following concentrations: ampicillin (Amp), 100 μg/ml; kanamycin (Kan), 50 μg/ml; cefoxitin (Cfx), 1 μg/ml; erythromycin (Erm) 1 μg/ml; and spectinomycin (Spec), 60 μg/ml.

### Growth assays on agar plates supplemented with different concentration of bovine hemoglobin

A 100 μl sample of an overnight culture (optical density at 600 nm [OD600] = 1) of the tested strain was mixed with 4 ml of soft agar and poured into LB plates. Wells were cut in the agar and filled with 150-μl aliquots of different concentrations of sterile bovine hemoglobin. Growth around the wells was recorded after 24 h or 48 h incubations at 37 °C.

### *In vitro* growth rate determination

The *in vitro* growth rates of the tested strains were determined by measuring the OD600 with a spectrophotometer (Eppendorf Biophotometer, Germany). Briefly, cultures in early exponential phase were inoculated in 20 ml TSB medium at OD600 = 0.1 and incubated at 37 °C with shaking at 180 rpm. The OD600 was determined every 2 h for 14 h.

### Conjugation

*E. coli* S17-1 strains harboring the tested plasmids were grown to early exponential phase in LB broth. *R. anatipestifer*, which is naturally resistant to Kan, was grown overnight on plates containing sheep blood at 37 °C and harvested by scraping. The bacteria were washed and re-suspended in 10 mM MgSO_4_. Then, the donor strain and the recipient strain were mixed at a ratio of 1:2 (2.5 × 10^8^:5 × 10^8^) and filtered through a 0.25-μM Millipore membrane. The membrane was incubated on a blood-containing agar plate at 30 °C for 8 h to 20 h. The filter was washed with 5 ml of 10 mM MgSO_4_, and 400 μl of bacterial suspension was spread onto blood-containing agar plates supplemented with Kan (20 μg/ml) and Cfx (1 μg/ml). The plates were incubated at 37 °C for 2 to 3 days.

### Construction of *E. coli–R. anatipestifer* shuttle vectors pLMF01, pLMF03, pLMF04 and pLMF05

*E. coli*–*R. anatipestifer* shuttle vectors were constructed based on the plasmid pMM47. A^27^ by replacing the replication regions in *Capnocytophaga canimorsus* with the putative replication region in the *R. anatipestifer* pRA0726 plasmid (Fig. S1). This plasmid served as an *E. coli–R. anatipestifer* shuttle vector and was designated pLMF01. The putative promoter sequences of the *B739_0921*, *B739_0973* and *B739_0889* plasmids, which have high, medium and low transcriptional activities, respectively, were amplified from RA-CH-1 using primers high exp P1 and high exp P2, medium exp P1 and medium exp P2, and low exp P1 and low exp P2 (Table S2). The PCR fragments were purified and digested with SalI and NcoI and ligated into the pLMF01 plasmid after its digestion with SalI and NcoI. The ligation mixtures were introduced into CaCl_2_-competent *E. coli* XL1-Blue cells, and transformants were selected on LB plates containing Amp (100 μg/ml final concentration). The presence of the correct inserts was confirmed by PCR and sequencing (BGI, Guangzhou, China). The created plasmids were designated pLMF03, pLMF04 and pLMF05 (Fig. S1).

### Shuttle plasmid stability assays

Vector stability assays were performed in *R. anatipestifer* as follows. The *E. coli–R. anatipestifer* shuttle vector pLMF01 was transferred into the *R. anatipestifer* ATCC11845 strain by conjugation. The recombinant *R. anatipestifer* strain RA ATCC11845::pLMF01 was grown on sheep blood-containing plates with or without antibiotics. After 10 generations, the *cfxA* gene of the pLMF01 vector was amplified by from 10 colonies. Additionally, plasmids prepared from these 10 colonies were subjected to PCR and restriction analysis.

### Construction of vectors for the complementation of RA ATCC11845 *∆tonB1*

The entire coding region of *tonB1* was PCR-amplified from RA ATCC11845 chromosomal DNA using the primers TonB1 Comp P1 and TonB1 Comp P2, containing NcoI and XbaI restriction sites, respectively (Table S2). The PCR product was purified, digested with NcoI and XbaI, and ligated into plasmids pLMF03, pLMF04 and pLMF05 (also digested with NcoI and XbaI) to generate plasmids pLMF03*::tonB1,* pLMF04*::tonB1,* and pLMF05*::tonB1*, respectively. The ligation products were introduced into *E. coli* strain XL1 blue cells using a calcium chloride method, and transformants were selected on LB plates containing Amp (100 μg/mL final concentration). The presence of the correct inserts was confirmed by PCR and sequencing (BGI, Guangzhou, China).

### Construction of plasmids pLMF03*::tonB2* and pLMF03*::tbfA*

The entire coding regions of *tonB2* and *tbfA* were PCR-amplified from RA ATCC11845 chromosomal DNA using primers TonB2 Comp P1 and TonB2 Comp P2 and primers TbfA Comp P1 and TbfA Comp P2, respectively. The P1 primers contained a NcoI restriction site, and the P2 primers contained a XbaI restriction site (Table S2). The PCR products were purified, digested with NcoI and XbaI, and ligated into pLMF03 plasmids digested with NcoI and XbaI to generate the plasmids pLMF03*::tonB2* and pLMF03*::tbfA.* The ligation products were introduced into *E. coli* strain XL1 blue cells using a calcium chloride method, and transformants were selected on LB plates containing Amp (100 μg/mL final concentration). The presence of the correct inserts was confirmed by PCR and sequencing (BGI, Guangzhou, China).

### Construction of plasmids for knockdown of TonB1 and TbfA

The *tonB1* and *tbfA* PCR products were amplified from *R. anatipestifer* ATCC11845 genomic DNA using primers TonB1-anti P1 and TonB1-anti P2 and primers TbfA-anti P1 and primer TbfA-anti P2, respectively. The P1 primers contained a XbaI restriction site, and the P2 primers contained an NcoI restriction site (Table S2). The PCR fragments were purified and digested with XbaI and NcoI. Then, the fragments were ligated into the pLMF03 plasmid, which was also digested with XbaI and NcoI. The ligation mixtures were introduced into CaCl_2_-competent *E. coli* strain XL1-Blue cells, and transformants were selected on LB plates containing Amp (100 μg/mL final concentration). After ligation into the vectors, the presence of the correct inserts was confirmed by PCR and sequencing (BGI, Guangzhou, China).

### DNA isolation, amplification, and electrophoresis

Kits and enzymes were used following manufacturer’s instructions. Small-scale plasmid DNA preparations were performed using a TIANprep Mini Plasmid Kit (TIANGEN, Beijing, China). Restriction, modification, and ligation were carried out according to manufacturer’s recommendations. DNA fragments were amplified in a Hybaid PCR thermocycler using Phusion DNA polymerase (NEB, Beijing, China). Agarose gel electrophoresis was performed using standard techniques. Purification of DNA fragments from PCR and restriction-digest reactions was accomplished using a Universal DNA Purification kit (TIANGEN, Beijing, China). The validity of all the fragments amplified by PCR was determined by sequencing (BGI, Guangzhou, China).

### Determination of plasmid copy number

The plasmid copy number was measured using the method provided by Lee *et al.*^17^. Briefly, The RA ATCC11845 cells harboring pLMF01 were cultured in TSB medium. Total DNA was extracted from the cultures during the exponential growth phase. The extraction was performed using the TIAamp Bacteria DNA Kit (TIANGEN), following a method described in the manufacturer’s instructions. The concentration of extracted DNA was measured using nanodrop 2000 spectrophotometer(Thermo). The prepared template DNA was analyzed to quantify *recA*, a single copy gene from RA ATCC11845 chromosomal DNA and *cfx*, a single-copy gene of plasmid pLMF01, in triplicate in real-time QPCR assay. The ratio of *cfx* to *recA* is equal to the plasmid copy number of pLMF01.

### Real-time PCR

The strains were inoculated at OD600 0.05 in 20 ml of TSB at 37 °C. After 6–8 h incubation (corresponding to the mid-log growth phase), the bacteria (~6 × 10^9^ CFU) was immediately mixed with 2-fold volumes RNAprotect Bacteria Reagent (Qiagen: 76506) and centrifuged again at 5000 g for 10 min. Bacteria were lysed in 200 μl TE buffer containing Proteinase K (60 mAU.ml^−1^, Qiagen: 19131) and Lysozyme (1 mg.ml^−1^, Sigma: L6876) for 10 min. RNA was extracted with the RNeasy Protect Bacteria Mini Kit (Qiagen: 74524) according to manufacturer instructions. To remove genomic DNA, an on-column DNase digestion and an additional DNase digestion post extraction were performed using RNAse-Free DNase set (Qiagen: 79254). Absence of genomic DNA was tested by PCR for *recA*. 800 ng of RNA were reverse transcribed using HiScript^TM^ reverse transcriptase and random/specific primers according to manufacturer instructions(Vazyme:R123-01). A no enzyme control was included for all RNA samples to confirm the absence of genomic DNA. qPCR was performed using SYBR Green Master Mix (Vazyme:Q111-01) and primers at 0.2 μM. Primers were designed with PerlPrimer software. Three samples and technical replicates were run for each target and condition. Before performing the actual qPCR, serial plasmid pLMF03*::tonB1* or pLMF03*::tbfA* dilutions were amplified and PCR and primer efficiencies were evaluated by means of a standard curve. All qPCR reactions were performed on a CFX Connect Real-time System (BIO-RAD) as recommended by the manufacturer. Fold change was calculated as described in ref. 28 with the delta delta Ct method considering the efficiency of the PCR reaction for each target. *recA* served as reference gene.

### Antibody Preparation

200 μl of an emulsion containing purified TbfA (50 μg) and freund’s adjuvant (100 μl) were inoculated twice (at half a month interval) celiac into 4 weeks old KunMing mice. Two weeks after the second inoculation, 200 μl blood samples were collected every 3 weeks via retro-orbital bleeding. Blood samples were centrifuged twice (3,600 rpm 5 min) to obtain serum which was stored at −20 °C. Before use, non-specific antibodies were removed by incubating the immune serum with *E. coli* cell extract for 1 h at 4 °C and centrifugation for 10 min at 8,000 rpm. The supernatant was then used as serum.

### Immunoblotting

SDS-PAGE and immunoblotting were used to detect TonB1 expression in *R. anatipestifer ∆tonB1* and *R. anatipestifer ∆tonB1* strains harboring a variety of plasmids. Verification of decreased expression in *R. anatipestifer* pLMF03::*tbfA-antisense* was performed as follows. The tested strains were collected, suspended in PBS buffer, and centrifuged at 5,000 rpm for 5 min. Bacterial pellets calculated to contain 20 μg of protein were suspended in loading buffer and heated for 5 min at 100 °C. Proteins were separated by 12% SDS-PAGE and subsequently transferred to a nitrocellulose membrane according to a standard protocol. Non-specific binding sites were blocked with 5% skim milk in TBS-Tween 20 (0.05%). The blot was probed with polyclonal mouse serum raised against recombinant TbfA or TonB1 (1:400) as described elsewhere^2^. Polyclonal mouse serum raised against recombinant RecA (1:400) was used as an internal reference as previously described^29^. Following this, the blot was probed with a 1:2,000 dilution of a goat anti-mouse IgG alkaline phosphatase-conjugated secondary antibody (CST). The binding of the antibodies to TbfA or TonB1 protein was revealed using a BCIP/NBT solution following manufacturer’s instructions (Sigma, China).

### Ethics Statement

Animals were handled in strict accordance with good animal practice as defined by the local animal welfare bodies. Animal work performed at the Sichuan Agriculture University was reviewed and approved by the Sichuan Agriculture University ethics committee on September 2015.

### Statistical Analysis

Statistical analysis was performed using GraphPad Prism 5 software for Windows. Statistical significance was ascertained using Student’s T test. P < 0.05 was considered significant.

### Nucleotide sequence accession number

The GenBank accession numbers for *R. anatipestifer* ATCC11845 and RA-CH-1 are CP003388.1 and CP003787.1, respectively. The sequences of the pLMF01, pLMF03, pLMF04 and pLMF05 plasmids reported have been deposited in GenBank under accession numbers KU963002, KU997673, KU997671 and KU997672.

## Additional Information

**How to cite this article**: Liu, M. F. *et al.* Investigation of TbfA in *Riemerella anatipestifer* using plasmid-based methods for gene over-expression and knockdown. *Sci. Rep.* **6**, 37159; doi: 10.1038/srep37159 (2016).

**Publisher’s note:** Springer Nature remains neutral with regard to jurisdictional claims in published maps and institutional affiliations.

## Supplementary Material

Supplementary Information
