# Supplementary material for: Investigation of TbfA in Riemerella anatipestifer using plasmid-based methods for gene over-expression and knockdown
Source: Sci Rep. 2016 Nov 15;6:37159. doi: 10.1038/srep37159 (PMC5109031; doi:10.1038/srep37159)
Supplement: Supplementary Information [file srep37159-s1.pdf]

Supplementary information

Investigation of TbfA in *Riemerella anatipestifer* using plasmid-based methods for gene over-expression and knockdown

MaFeng Liu[1,2,3+*], MengYi Wang[1,2,3+], DeKang Zhu[2,3], MingShu Wang[1,2,3], RenYong Jia[1,2,3], Shun Chen[1,2,3], KunFeng Sun[1,2,3], Qiao Yang[1,2,3], Ying Wu[1,2,3], XiaoYue Chen[1,2,3], Francis Biville[4], AnChun Cheng[1,2,3*]

1. Institute of Preventive Veterinary Medicine, Sichuan Agricultural University, Chengdu, Sichuan 611130, P.R. China

2. Avian Disease Research Center, College of Veterinary Medicine of Sichuan Agricultural University, Chengdu, Sichuan 611130, P.R. China

3. Key Laboratory of Animal Disease and Human Health of Sichuan Province, Sichuan Agricultural University, Chengdu, Sichuan 611130, P. R. China

4. Unité des Infections Bactériennes Invasives, Département Infection et Epidémiologie, Institut Pasteur, Paris, France

[+]These authors contributed equally to this work.

* Corresponding author:

E-mail: liumafengra@163.com (MF Liu), chenganchun@vip.163.com (AC Cheng)

Supplementary Figures

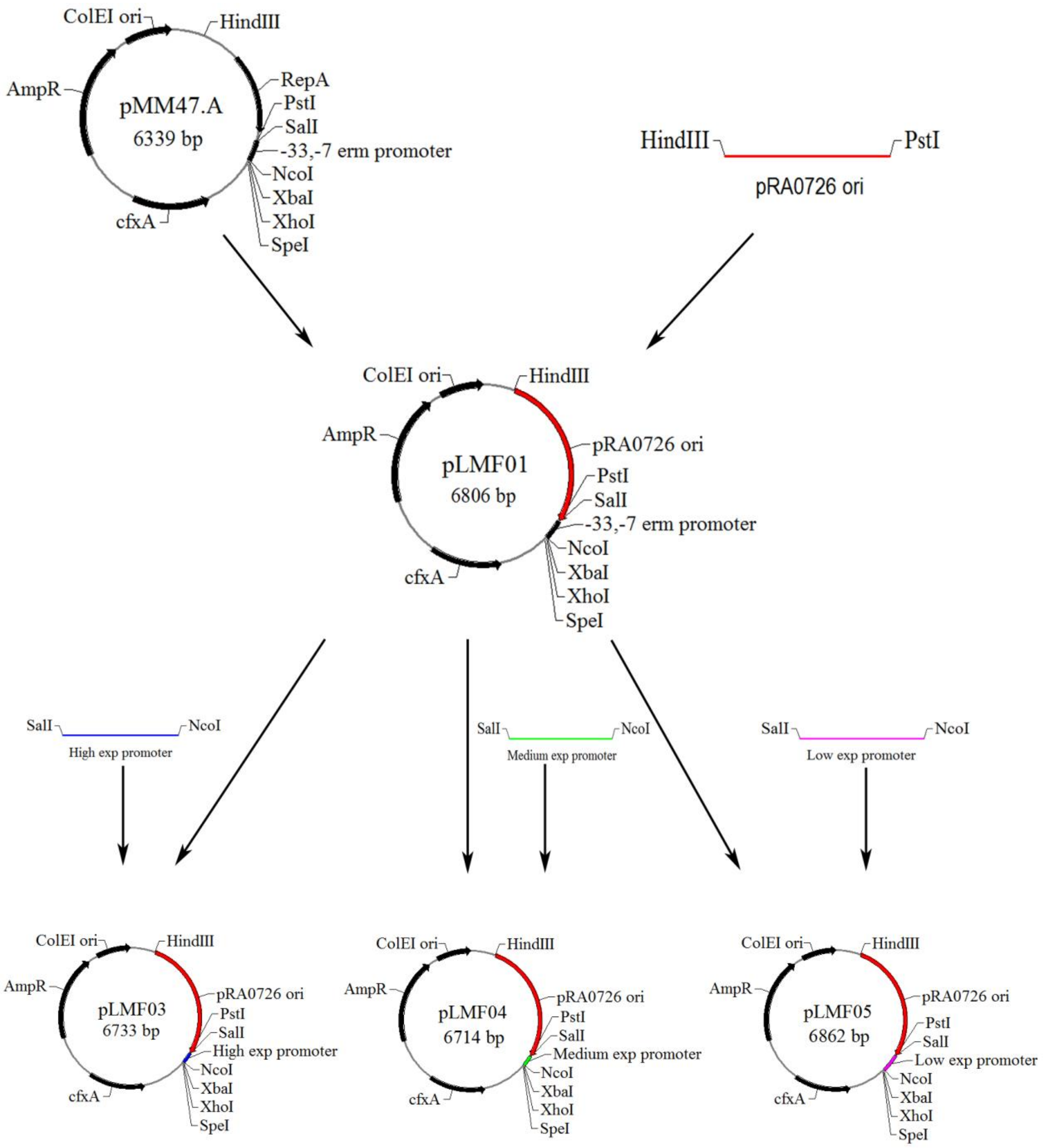

Supplementary Figure S1. Construction of the shuttle vectors pLMF01, pLMF03, pLMF04 and pLMF05. Vectors containing the *cfxA* gene (Cfr) for selection in *R. anatipestifer* and the replication region of plasmid pRA0726 were designed. The promoter region of B739_0921 was used to create the pLMF03 plasmid. The promoter of B739_0973 was used to create the pLMF04 plasmid. The promoter region of B739_0889 was used to create the pLMF05 plasmid. The vectors contained the replication origin for *E. coli* and an ampicillin resistance gene upstream from NcoI,

XbaI, XhoI and SpeI restriction sites, which allowed the insertion of the desired coding sequences.

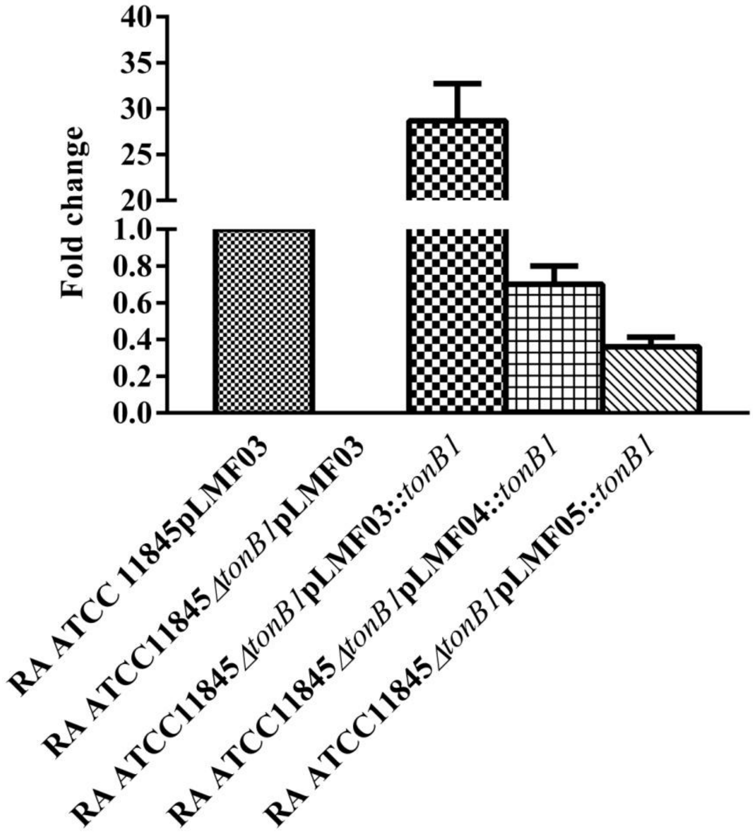

Supplementary Figure S2. The transcription level of *tonB1* gene under different promoters. Figure shown are average fold differences in the mRNA quantities of *tonB1* gene in strain RA ATCC11845*ΔtonB1*pLMF03*::tonB1*, RA ATCC11845*ΔtonB1*pLMF04*::tonB1* and RA ATCC11845*ΔtonB1*pLMF05*::tonB1*, relative to the quantities of RA ATCC11845pLMF03. The amount of transcription *tonB1* gene mRNA from each strain was normalized to the amount of *recA*. Error bars represent standard deviations from three independent triplicate determinations.

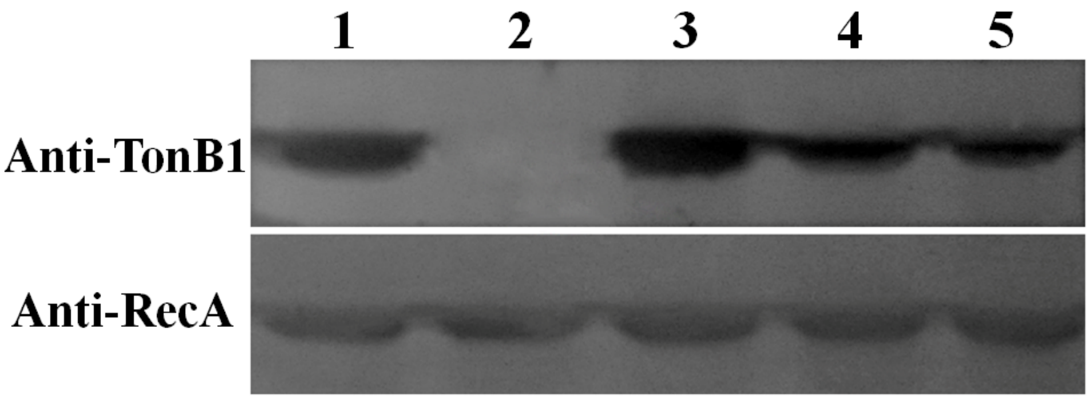

Supplementary Figure S3. Immunoblotting to detect TonB1 expression levels produced by shuttle vectors with different promoters. TonB1 expression levels were detected in *R. anatipestifer* ATCC11845*ΔtonB1* strains harboring different shuttle plasmids via immunoblot analysis using an anti-TonB1 antibody. To guarantee equal sample loading, the RecA protein was used as a reference protein. *R. anatipestifer* ATCC11845*ΔtonB1* strains containing plasmids with various promoters are shown as follows. Lane 1: *R. anatipestifer* ATCC11845pLMF03, lane 2: *R. anatipestifer* ATCC11845*ΔtonB1*pLMF03, lane 3: *R. anatipestifer* ATCC11845*ΔtonB1*pLMF03 *::tonB1,* lane 4: *R. anatipestifer* ATCC11845*ΔtonB1*pLMF04 *::tonB1,* lane 5: *R. anatipestifer* ATCC11845*ΔtonB1*pLMF05 *::tonB1*. Measurement of TonB1 band intensity using Image J software gave the following results. Lane 1: mean integrated density, 44; lane 2: mean integrated density, 2; lane 3: mean integrated density, 58; lane 4: mean integrated density, 42; lane 5: mean integrated density, 34. Measurement of RecA band intensity using Image J software gave the following results. Lane 1: mean integrated density, 20; lane 2: mean integrated density, 21; lane 3: mean integrated density, 22; lane 4: mean integrated density, 21; lane 5: mean integrated density, 20.

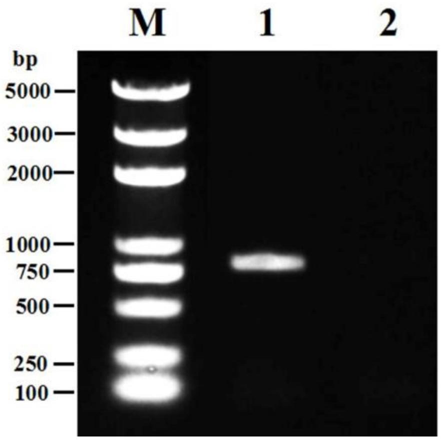

Supplementary Figure S4. Identification of antisense transcript in RA ATCC11845 pLMF03*::tbfA-antisense* (Lane 1) and RA ATCC11845 pLMF03(Lane 2). Total RNA was extracted from the strain ATCC11845 pLMF03*::tbfA-antisense* and RA ATCC11845 pLMF03, respectively. 800 ng of RNA were reverse transcribed using specific primer TbfA antiRTP2(Table S2) according to the method described in the *Materials and Methods* section. PCR was performed using the primer TbfA Comp P1 and TbfA Comp P2(Table S2).

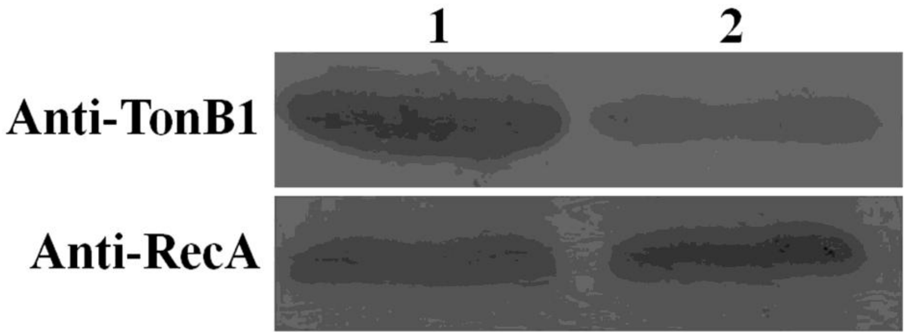

Supplementary Figure S5. Immunological analysis of TonB1 expression in RA ATCC11845 pLMF03 (control) and TonB1 knockdown strains. Each strain was collected from overnight cultures grown on blood-containing plates. All lanes were loaded with equivalent amounts of total protein based on the OD600 of the culture. After SDS-PAGE, the samples were transferred onto membranes to perform western blotting using an antibody against TonB1. Percentage reductions in protein levels were calculated by quantitatively analyzing the TonB1 bands on the western blots. Measurement of TonB1 band intensity using Image J software gave the following results. Lane 1: integrated density, 42; lane 2: integrated density, 19. Measurement of RecA band intensity using Image J software gave the following results. Lane 1: integrated density, 18; lane 2: integrated density, 19.

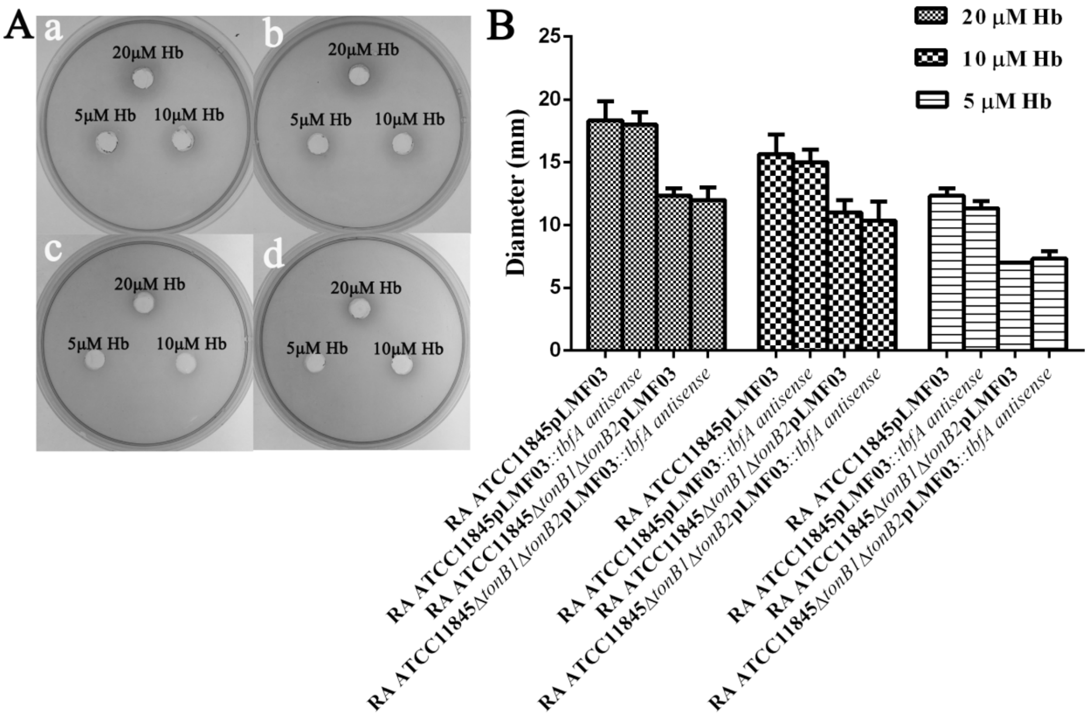

Supplementary Figure S6. Hemin transport activity assay for RA ATCC11845 pLMF03 (a), RA ATCC11845 pLMF03*::tbfA-antisense* (b), RA ATCC11845 Δ*tonB1*Δ*tonB2* pLMF03 (c), and RA ATCC11845 Δ*tonB1*Δ*tonB2*pLMF03*::tbfA-antisense* (d). The above strains were tested for hemin utilization efficiency on LB plates as described in the *Materials and Methods* section. After 24 h or 48 h of growth, the diameter of the zone of turbidity in each well was measured in quadruplicate for each plate, and the mean diameter was calculated. The results are expressed as the mean ±SD of the diameters (in mm) obtained for the three plates. The data were analyzed using Student’s t-test. There were no significant differences among the measurements.

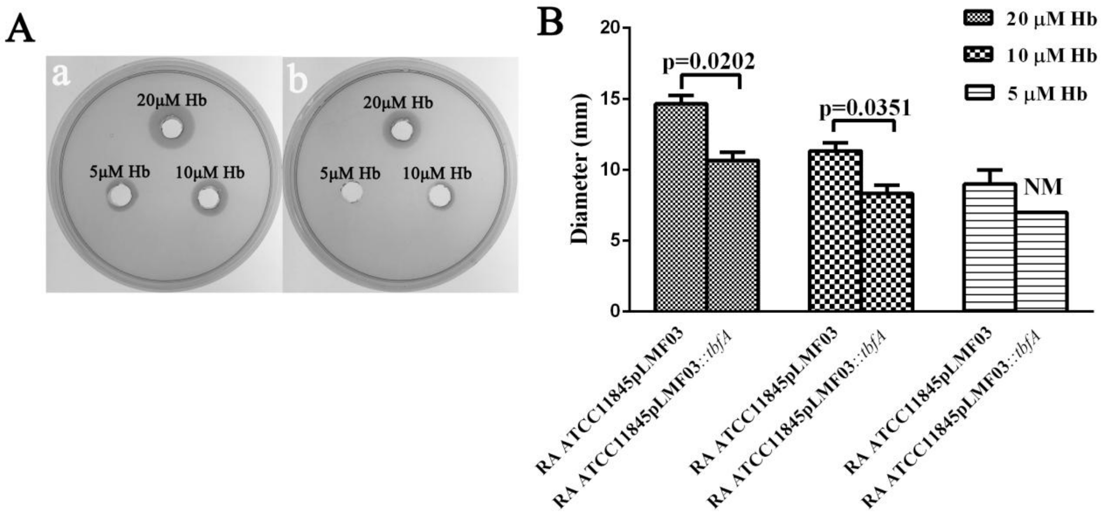

Supplementary Figure S7. Hemin transport activity assay for RA ATCC11845pLMF03(a) and RA ATCC11845pLMF03::*tbfA*(b). The above strains were tested for hemin utilization efficiency on LB plates as described in the *Materials and Methods* section. After 24 h of growth, the diameter of the zone of turbidity in each well was measured in quadruplicate for each plate, and the mean diameter was calculated. The results are expressed as the mean±SD of the diameters (in mm) obtained for the three plates. NG: No growth in the well. The data were analyzed using Student’s t-test.

Supplementary Tables

Supplementary Table S1. Strains and plasmids used in this study.

***E. coli* strains**	**Genotype**	**Source or reference**
XL1-BLUE	F^- *supE44 hdsR17 recA1 endA1 gyrA46 thi relA1* lac^- F' *proAB*$^-$ *lacI*^{q} *lacZΔM15* Tn*10*, Tet	Laboratory collection
S17-1	*hsdR17 recA1* RP4-2-tet ::Mu-1kan::Tn7 ; Sm^R	[1]
***Riemerella anatipestifer* strains**	**Genotype**	**Source or reference**
R. anatipestifer ATCC11845	ATCC11845	[2]
R. anatipestifer CH-1	Seroytype 1	[3]
R. anatipestifer CH-2	Seroytype 2	[3]
R. anatipestifer ATCC11845*Δtonb1*	ATCC11845*Δtonb1*,SpcR	[4]
R. anatipestifer ATCC11845*Δtonb1Δtonb2*	ATCC11845*Δtonb1Δtonb2*, SpcR,ErmR	[4]
**Plasmids**	**Genotype**	**Source or reference**
pMM47.A	*ermF* promoter, *ori*ColE1, *ori* pCC7, Ap^R, Cfx^R.	[5]
pLMF01	*ermF* promoter, *ori*ColE1, *ori* pRA0726, Ap^R, Cfx^R	This study
pLMF03 .	B739_0921 promoter, *ori*ColE1, *ori* pRA0726, Ap^R, Cfx^R	This study
pLMF04	B739_0973 promoter, *ori*ColE1, *ori* pRA0726, Ap^R, Cfx^R	This study
pLMF05	B739_0889 promoter, *ori*ColE1, *ori* pRA0726, Ap^R, Cfx^R	This study
pLMF03::*tonb1*	pLMF03 carrying *tonB1* from *R. anatipestifer* ATCC11845, Ap^R, Cfx^R	This study
pLMF04::*tonb1*	pLMF04 carrying *tonB1* from *R. anatipestifer* ATCC11845, Ap^R, Cfx^R	This study
pLMF05::*tonb1*	pLMF05 carrying *tonB1* from *R. anatipestifer* ATCC11845, Ap^R, Cfx^R	This study
pLMF03::*tonb2*	pLMF03 carrying *tonB2* from *R. anatipestifer* ATCC11845, Ap^R, Cfx^R	This study
pLMF03::*tonbf*	pLMF03 carrying *tonBf* from *R. anatipestifer* ATCC11845, Ap^R, Cfx^R	This study
pLMF03::*tonb1antisense*	pLMF03 carrying antisense *tonB1* from *R. anatipestifer* ATCC11845, Ap^R, Cfx^R	This study
pLMF03::*tonbf antisense*	pLMF03 carrying antisense *tonBf* from *R. anatipestifer* ATCC11845, Ap^R, Cfx^R	This study

AmpR, ampicillin resistance; KmR, kanamycin resistance; ErmR, erythromycin resistance; SpcR, spectinomycin resistance.

Supplementary Table S2. Primers used in this study.

Primer	Organism	Sequence
RA Rep P1:	pUC57::AB0117Ori(RA)	CCCAAGCTTGGGCTATTTAGGCATTAGCCCTC
RA Rep P2	pUC57::AB0117Ori(RA)	AAAACTGCAGCCAATGCATTGGAACAGATCTCGTATAGAGCTCG
cfxA P1	pMM47.A	GGTGCTGCAATGTTGATG
cfxA P2	pMM47.A	CCGCTAAGGTATAACTG
Low exp P1	RA-CH-1	ACGCGTCGACGTCGGCCATAGAAATCACCAATGATTGGTAC
Low exp P2	RA-CH-1	CATGCCATGGCATGTTTATAAGAAACTTTAAATTAAAAATAAATTACC
Medium Exp P1	RA-CH-1	ACGCGTCGACGTCGGCCATAGCGGATTAGTAGGTACTCAG
Medium Exp P2	RA-CH-1	CATGCCATGGCATGAATTTTATATATTAGATGTTAGATGTTAG
High Exp P1	RA-CH-1	ACGCGTCGACGTCGGCCATATTTCAAAAATTTAACTTAAACC
High Exp P2	RA-CH-1	CATGCCATGGCATGAATTTTAAATAATTTTTTTAAATTTG
TonB1 Comp P1	RA-ATCC	CATGCCATGGATGAGCCAAACCATAAATAC
TonB1 Comp P2	RA-ATCC	CTAGTCTAGATTAGAAAGTGATTTTGTAAGTGCCTG
TonB2 Comp P1	RA-ATCC	CATGCCATGGATGTCAGATGAAAATTTAGGGTACAATCC
TonB2 Comp P2	RA-ATCC	GCTCTAGAGCTTAATACTCAAAATTCATTGCCACTGGC
TbfA Comp P1	RA-ATCC	CATGCCATGGATGATAACTTTTTCACAAAACCAAAACG
TbfA Comp P2	RA-ATCC	CTAGTCTAGATCATTCAAACTTTATAGATACAGG
TonB1-antis P1	RA-ATCC	CTAGTCTAGAATGAGCCAAACCATAAATAC
TonB1-antis P2	RA-ATCC	CATGCCATGGTTAGAAAGTGATTTTGTAAGTGCCTG
TbfA-antis P1	RA-ATCC	CTAGTCTAGAATGATAACTTTTTCACAAAACCAAAACG
TbfA-antis P2	RA-ATCC	CATGCCATGGTCATTCAAACTTTATAGATACAGG
TonB1 qRTP1	RA-ATCC	AAAGGAGGAAGTCGTAAGC
TonB1 qRTP2	RA-ATCC	TGAGGTTCTACAGGTGTAGG
cfxA qRTP1	pLMF01	AGAGTGACAACAATGCAAGC
cfxA qRTP2	pLMF01	CGATTCATCAACATTGCAGC
RecA qRTP1	RA-ATCC	TGAAACTAGGTGATGGTACG
RecA qRTP2	RA-ATCC	CTTAGGATAACCGCCTACTC
TbfA qRTP1	RA-ATCC	ATGAGCTCTTATTTGCGGG
TbfA qRTP2	RA-ATCC	CTCCAAATACAGCTACTCCTG
TbfA antiRTP2	RT-ATCC	GATAACTTTTTCACAAAACC

Reference:

1 Simon, R., Priefer, U., P, A., uuml & hler. A Broad Host Range Mobilization System for In Vivo Genetic Engineering: Transposon Mutagenesis in Gram Negative Bacteria. *Nature Biotechnology* **1**, 784-791,(1983).

2 Wang, X. *et al.* Complete genome sequence of Riemerella anatipestifer reference strain. *Journal of bacteriology* **194**, 3270-3271,(2012).

3 Wang, X. *et al.* Comparative genomics of Riemerella anatipestifer reveals genetic diversity. *BMC genomics* **15**, 479,(2014).

4 Liao, H. *et al.* TonB Energy Transduction Systems of Riemerella anatipestifer Are Required for Iron and Hemin Utilization. *PloS one* **10**, e0127506,(2015).

5 Mally, M. & Cornelis, G. R. Genetic tools for studying Capnocytophaga canimorsus. *Applied and environmental microbiology* **74**, 6369-6377,(2008).
